# Mental health literacy interventions for female adolescents: a systematic review and meta-analysis

**DOI:** 10.1007/s00787-025-02648-2

**Published:** 2025-01-22

**Authors:** Emily R. Arnold, Caitlin Liddelow, Angie S. X. Lim, Stewart A. Vella

**Affiliations:** Global Alliance for Mental Health and Sport (GAMeS), School of Psychology, Faculty of the Arts, Social Sciences and Humanities, University of Wollongong, Northfields Avenue, Wollongong, NSW 2522 Australia

**Keywords:** Adolescent girls, Knowledge, Stigma, Help-seeking, Effectiveness, Review

## Abstract

Suicide and self-harm are a leading cause of death globally, with females aged 15–24 years being a high-risk group requiring urgent intervention. Promoting mental health literacy is a well-established strategy for early intervention. No review has explored the available interventions promoting mental health literacy for female adolescents and their reach, effectiveness, adoption, implementation, and maintenance as a whole remains unknown. This study reviews existing interventions promoting mental health literacy in female adolescents. Searches included original peer-reviewed articles from inception to May 2024 across six databases, identifying twelve studies for review. Five independent meta-analyses were conducted, showing no significant effects immediately post-intervention for mental health literacy, knowledge, stigmatising attitudes, and help-provision. A significant reduction in stigmatising attitudes was found > 6 months post-intervention. Moderation analyses could not be performed due to insufficient data. None of the included studies reported on interventions specifically for female adolescents. This review also evaluated reporting of RE-AIM components, finding limited reporting on reach, adoption, and maintenance. These findings highlight a substantial gap in high-impact and tailored mental health prevention and promotion interventions for female adolescents. Therefore, there is a clear need to understand and target female adolescents’ mental health literacy needs to develop and implement more effective interventions.

**Supplementary Information:**

The online version contains supplementary material available at 10.1007/s00787-025-02648-2.

## Introduction

Adolescent mental health disorders are recognised as one of the leading contributors of disease burden in young people [1]. Adolescence (ages 10–19 years, [2]) is a stage of development whereby the psychological and physical transition from childhood to adulthood brings unique challenges [3]. These challenges can include emerging mental illness, identity exploration, educational achievement, and social relationships [4]. Experiencing these challenges puts adolescents at increased risk of developing mental health disorders, with half of mental health problems having their onset before the age of 14 [5]. Globally, 12.4% of adolescents aged 10–14, 13.96% aged 15–19, and 13.63% aged 20–24 experience a mental health problem annually [6], with suicide ranking as the fourth leading cause of death worldwide [7] and the leading cause of death in Australia for these age groups [8]. Female adolescents represent the group at highest risk of psychological distress, lower life satisfaction, self-harm, and suicidal behaviour (e.g., considering attempting suicide), compared to male adolescents [9–12]. The distress and impairment associated with these mental health problems often persists into adulthood [13], and if ignored, can profoundly shape the course of emotional, behavioural, and psychological functioning and quality of life in later years [14]. Despite these consequences, approximately 40% of female adolescents who experience mental health problems do not access professional help [15], with financial cost, stigma, negative beliefs and attitudes, and low mental health literacy recognised as major barriers [16, 17].

Research has demonstrated key differences in the prevalence and clinical presentations of mental health problems and suicide between male and female adolescents [18, 19]. These differences can be attributed to ‘sex’ and ‘gender’ [18]. Sex refers to the biological classification of male and female according to chromosomes, hormones, and reproduction. Gender refers to one’s self-representation influenced by psychosocial and cultural factors [20]. Explained by the biopsychosocial model [21], biological and psychosocial factors associated with sex and gender may lead to different mental health vulnerabilities and resiliencies. Specifically, biological factors such as genetics, hormonal fluctuations, and neurotransmitters may predispose individuals to mental health problems, potentially influencing their vulnerability to specific mental health conditions [22]. For example, developing during puberty, females may be more susceptible to internalising problems such as anxiety or depression due to earlier pubertal development [23, 24] whereas males may be more susceptible to externalising problems such as conduct disorder due to higher testosterone levels [25]. Additionally, psychosocial factors such as cognitive, affective, behavioural, and sociocultural expectations associated with gender dictate norms prescribing and proscribing individuals’ thoughts, feelings, and behaviours [26]. For example, females who internalise conventional femininity (e.g., body objectification and complying with interpersonal relationships) have shown to experience higher levels of depressed mood and low self-esteem [27], which may mediate the association with suicidal thoughts and behaviours [28]. Given the magnitude of these gender differences in mental health presentations and experiences, it is critical to explore the factors specific to female adolescents to better understand and improve engagement with and access to mental health care.

Low mental health literacy, characterised by a lack of knowledge and resistance to help-seeking, creates significant barriers to accessing mental health services [29]. Mental health literacy was first conceptualised by Jorm et al. [30, p.182] and was defined as “knowledge and beliefs about mental disorders which aid their recognition, management, or prevention”. More recently, mental health literacy has been redefined to include knowledge of mental health problems and corresponding treatments, understanding how to support positive mental health, reducing stigma surrounding mental health challenges, and improving help-seeking and self-help management strategies [31, 32]. This multifaceted definition provides a foundation for mental health decision making and addresses key factors that influence the trajectory of mental health [32, 33]. For example, an adolescent who recognises the signs and symptoms of a mental health disorder will more likely engage in adaptive help-seeking practices [34]. To raise awareness of the mental health needs of young people, international and national recommendations promote mental health literacy as a strategy for early intervention, promotion of positive mental health and suicide prevention [35–37]. While female adolescents have been shown to have higher mental health literacy than males [38], the National Women’s Health Strategy 2020–2030 still note it as a critical area of improvement [39]. As such, the development of mental health literacy could facilitate mental well-being and suicide prevention for female adolescents [40].

Previous research suggests that mental health literacy can be increased through targeted interventions [41], with psychosocial and educational interventions being the most common [42]. Reviews have explored the impact of interventions currently available to address poor mental health literacy and encourage help-seeking in adolescents [43–46]. From these studies, school and community-based interventions were likely to improve mental health related knowledge, attitudes, and help-seeking behaviour and reduce mental health stigma. Therefore, the use of these programs can protect adolescents against mental health problems and suicidality [47], and alleviate the physical, social, and financial burden associated with these challenges [48]. However, these reviews treated adolescents as a homogeneous collective, inadequately considering gender disparities in this domain.

### The current study

Given the magnitude of gender differences in adolescent mental health profiles, catering for these needs in mental health care is essential in developing effective programs [49]. Though, to our current knowledge, no review has been conducted to explore the currently available interventions promoting mental health literacy for female adolescents. Evaluating existing interventions allows us to recognise and understand effective strategies and areas of improvement in addressing the distinct mental health needs of female adolescents. Therefore, the primary aim of this study is to systematically review and meta-analyse the existing interventions promoting mental health literacy in female adolescents for suicide prevention. In doing so, we seek to understand their purpose, reach, effectiveness, adoption, implementation, maintenance, potential theoretical foundations, and why female adolescents find the current programs effective or otherwise.

## Methods

This review protocol was pre-registered with the Centre for Reviews and Dissemination (PROSPERO; CRD42023418585) and followed the Preferred Reporting Items for Systematic Reviews and Meta-Analyses guidelines (PRISMA; [50, 51]).

### Search strategy

To find all available studies reporting on interventions promoting mental health literacy in female adolescents, six electronic databases were searched through EBSCOhost: (a) PsycInfo; (b) PsycArticles; (c) CLINHAL Plus; (d) MEDLINE; (e) Sport Discus; and (f) Scopus. Each database was searched from its year of inception to the search date of May 16, 2023, and then updated on May 27, 2024. Search limiters such as the English language and peer-review were applied. Truncation, wildcards, and key words were used and combined with Boolean operators (e.g., ‘OR’ ‘AND’). All fields were applied at the title and abstract level to maximise result relevance. See Table 1 for the search strategy used across all databases.

**Table 1:** Search terms and strategy used

1. AB OR TI “mental health literacy” OR “mental health knowledge” OR “mental health learning” OR “mental health awareness” OR “psycholog* literacy” OR “psycholog* knowledge” OR “psycholog* learning” OR “psycholog* awareness”
2. AB OR TI interven* OR program* OR “randomi#ed control* trial*” OR “RCT” OR trial* OR evaluat*
3. AB OR TI girl* OR female OR wom#n
4. AB OR TI teen* OR youth OR adolescen* OR “young people” OR “young person*”
5. S1 AND S2 AND S3 AND S4

*AB* abstract, *TI* title, *Limiters* English language, peer-reviewed

### Eligibility criteria

To meet inclusion criteria, studies had to: (a) be published in the English language; (b) be original research and published in peer-reviewed journals; (c) include female adolescents aged from 10–19 years [2]; (d) implemented or evaluated a mental health literacy intervention in adolescent females; and (e) assessed mental health literacy or associated components as the main outcome variables. There were no restrictions on study design (e.g., randomised control trial, pre-post designs, qualitative, mixed methods) or on the setting, length, or facilitator of the intervention. Inclusion was restricted to peer-reviewed literature only as unpublished or grey literature (e.g., dissertations, theses, reports) varies widely in quality and there is little guidance on the systematic methodology of reviewing this work [52]. Studies were excluded if they: (a) included diagnostic and treatment-based interventions due to their clinical and therapeutic approach (e.g., Cognitive Behavioural Therapy; [53]); (b) were reviews or secondary in nature (e.g., systematic reviews; [54]); (c) included male and female participants, wherein female data could not be independently extracted (e.g., [55]).

### Screening process

All studies were exported into Covidence [56] from their respective databases. Following the removal of duplicates, studies were independently screened at the title and abstract level by two authors (EA and AL). There was an agreement rate of 79% (Cohen’s *k* = 0.29) at this stage. Once full texts were located for relevant studies, two authors (EA and AL) reviewed the full texts in accordance with eligibility criteria, with an agreement rate of 85% (Cohen’s *k* = 0.55). Any discrepancies were solved through discussion between the two screeners. Reference lists of included studies were also searched to locate additional relevant studies that may have been missed in the original database searches. Corresponding authors were contacted to provide further information about their studies for inclusion where necessary. If no response was received from the author, the study was subsequently excluded from any relevant analyses.

### Data extraction

One author (EA) extracted data from the included studies using a data extraction table in Microsoft Excel, created specifically for this review. The data extraction excel was piloted prior to formal use to ensure suitability for the range of studies included in the review. Detailed descriptive information including publishing information (e.g., title, authors, year of publication, country); sample characteristics (e.g., sample size, mean age, country); study design features (e.g., study design, data collection points, control measures [e.g., waitlist, treatment as usual]); and intervention characteristics (e.g., name, context, purpose, length, mode of delivery, facilitator details, content) were extracted. Descriptive and statistical data from included studies were extracted as guided by the RE-AIM framework [57]. Consisting of five domains (Reach [e.g., measure of participation], Effectiveness [e.g., impact of intervention], Adoption [e.g., number, proportion and representativeness of settings], Implementation [e.g., delivery as intended], and Maintenance [e.g., sustained intervention impacts]), RE-AIM can be used to evaluate public health interventions, including those targeting mental health literacy (e.g., [58]). Addressing these domains in reviewing current mental health literacy interventions provides a comprehensive and practical structure of assessment for replicability, adaptivity and feasibility [59].

To assess intervention effectiveness, main outcomes and reported values for effectiveness (e.g., Cohen’s *d*) and standard error were manually calculated and extracted. Studies with missing statistical information were excluded from the meta-analysis if they could not be manually calculated or if they were not provided by the author upon request. When multiple effect sizes were reported under the same outcome domain, they were averaged and used within the meta-analysis (see [60, 61] for similar procedures). Narrative findings, if reported, were also extracted, and categorised according to the RE-AIM framework. Lastly, the third author (AL) cross-checked a random 20% (approximately *n* = 3) of extracted data from included studies for accuracy. The meta-analyses and the RE-AIM framework are based on female data only to explore the extent that female adolescents are represented in the literature.

### Study quality

The included studies were critically appraised using the Joanna Briggs Institute (JBI) Critical Appraisal Tools [62–64] and the National Heart, Lung, and Blood Institute (NHLBI) assessment tool Quality Assessment Tool for Observational Cohort and Cross-Sectional Studies [65]. Adding to the protocol, the Mixed Methods Appraisal Tool (MMAT) Version 2018 was included to account for mixed method studies [66]. One member of the research team (EA) assessed each study included for data synthesis. A second member of the research team (AL) assessed a random 20% (approximately *n* = 3) of included studies to ensure accuracy. No discrepancies were present during study quality checks. Using cut off scores to determine whether a study is low, moderate, or high quality is generally advised against as these responses do not give an accurate representation of specific problems of a study [66]. As such, no studies were excluded based on their quality or risk of bias.

### Synthesis of findings

To determine intervention effectiveness, statistical syntheses (i.e., meta-analyses) were performed with eligible studies to address effectiveness of RE-AIM. A narrative synthesis using RE-AIM categorisation was then conducted to summarise, explore, and integrate key findings. An extraction tool was modified from earlier RE-AIM reviews [67, 68] and included 34-items to understand and evaluate RE-AIM outcomes. Importantly, the syntheses based on the RE-AIM framework and the meta-analyses are based on female data only.

### Meta-analyses

A meta-analysis was performed using JASP v0.19.3 [69] using data from post-intervention and > 6 month follow up, with the estimation based on the restricted maximum likelihood estimator. For combining and reporting the results, each study’s outcomes were inspected and categorised in accordance with key mental health literacy constructs [32]: mental health literacy overall, knowledge of mental health, stigmatising attitudes, and help provision. Separate meta-analyses were conducted for each construct. A standard random-effects model was chosen as it is the most accurate estimation of the pooled effect size when effect sizes are different between studies (heterogeneity) [70]. *Q* and *I*^*2*^ statistics were used to assess the degree of heterogeneity, with *Q* indicating statistical significance and *I*^*2*^ indicating the percentage of variation across studies. *I*^*2*^ statistics of 25%, 50% and 75% are considered low, moderate, and high values of heterogeneity, respectively [71]. Effect sizes were interpreted as small (*d* = 0.20), medium (*d* = 0.50), and large (*d* = 0.80) [72]. Moderation analyses were planned to be conducted to explore possible sources of heterogeneity, however, the minimum number of studies for continuous (6–10 studies) and categorical (4 studies) subgroups was not achieved [73]. Therefore, moderation analyses could not be performed. The risk of publication bias was not formally assessed due to the exclusion of unpublished or grey literature and the limited number of studies in each meta-analysis (fewer than 10), reducing the power of tests to detect asymmetry [74, 75]. However, funnel plots were visually inspected for exploratory purposes (see Supplementary File 1).

### Reach

Reach was assessed using five criteria including participant exclusion criteria, participation rate (i.e., percent of individuals who participate based on a valid denominator), characteristics of participants in comparison to eligible non-participants, and use of qualitative methods to understand reach or recruitment outcomes. Methods to identify the target population was added as an additional criterion.

### Effectiveness

To explore effectiveness, six indictors were used, including primary outcome measures (e.g., mental health literacy), broader outcome measures (e.g., quality of life), measures of robustness (e.g., moderation analysis), short-term attrition (e.g., program completion or short-term follow-up), and use of qualitative methods to understand outcomes. Intention to treat analysis was added as an additional criterion.

### Adoption

Adoption indicators (eight criteria) were assessed at both the setting and staff level. Specifically, studies were reviewed to identify the extent to which they addressed setting exclusions, percentage of settings approached that participated, and characteristics of participating settings compared to eligible non-participating settings. Further, staff exclusions, percentage of invited staff participating, characteristics of staff participating compared to eligible non-participating staff, and use of qualitative methods were also considered.

### Implementation

The extent to which studies addressed intervention implementation were assessed using six criteria. This included any reported adaptations made to the intervention, the extent the protocol was delivered as intended, cost of delivery, consistency in implementation across staff, settings and subgroups, and use of qualitative methods. The theoretical basis of the interventions was also included as an additional criterion.

### Maintenance

At the individual level (five criteria), maintenance was determined by the assessment of intervention outcomes (primary [e.g., mental health literacy] and broader outcomes [e.g., quality of life, wellbeing]) at six or more months following the completion of individual participation, robustness of data, long-term attrition rates, and use of qualitative methods to understand long term impacts. At the organisational level (four criteria), maintenance was determined by the extent to which the program was sustained six months post study, if and how the intervention was adapted long-term, comments on sustainability of the intervention, and use of qualitative methods to understand setting level institutionalisation.

## Results

### Study selection

**Fig. 1:**
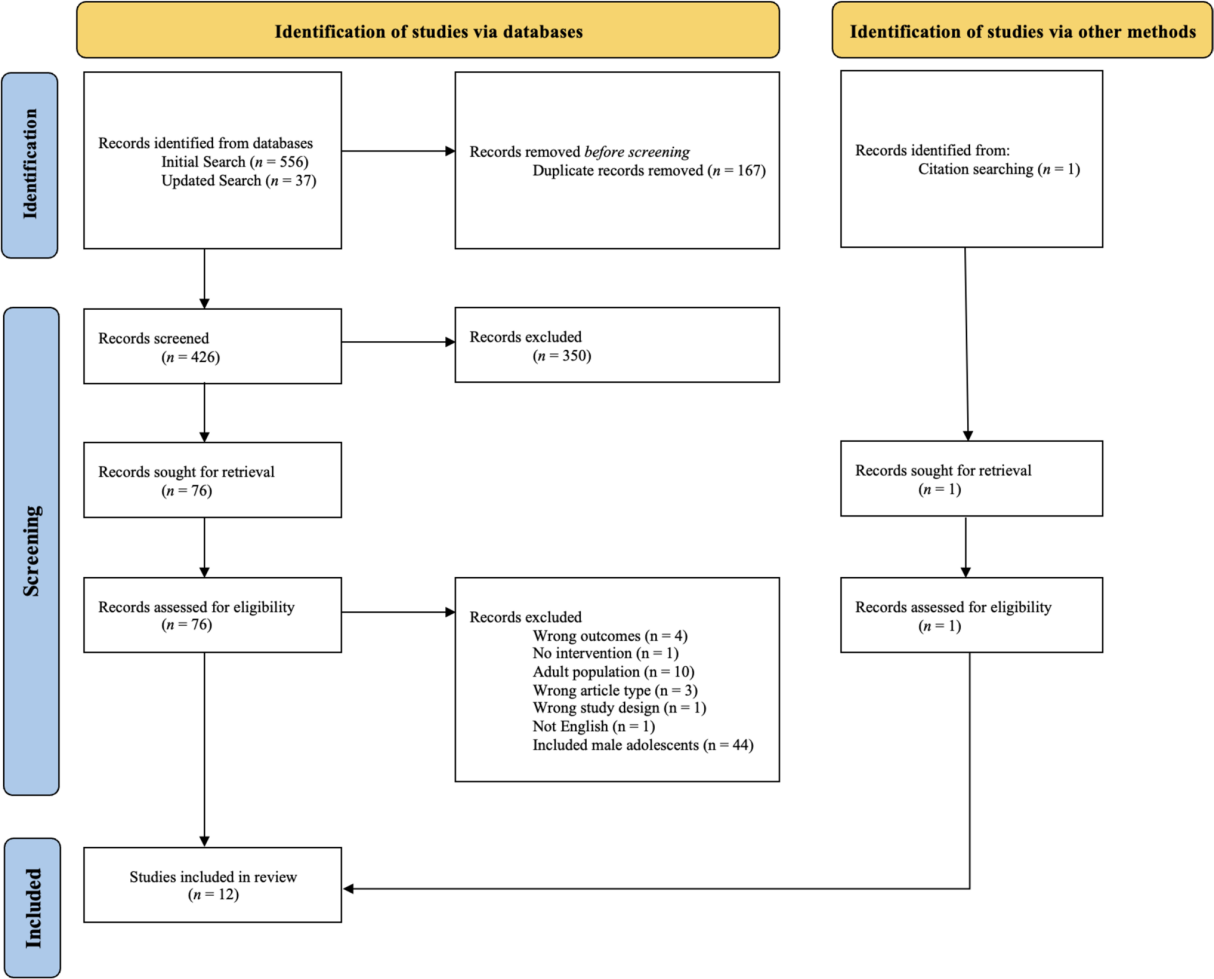
PRISMA 2020 diagram

From the initial electronic database searches, a total of 556 published studies were identified. The first author conducted an updated search in May 2024 using the same search criteria. However, the date range only included studies published after the first search date, which identified an additional 37 published studies (total *n* = 593). After duplicates were removed from both searches (total *n* = 168), the remaining 425 studies were screened against the inclusion and exclusion criteria. Of these, *n* = 350 studies were excluded from title and abstract screening, resulting in *n* = 75 studies for full-text screening. Following the full-text stage, 11 studies were included in this review. Further, one additional study was identified through reference searches of the included full-text studies, resulting in a total of 12 individual studies in this review. The PRISMA diagram summarising the flow of studies is provided in Fig. 1.

### Study characteristics

The included studies were published between 2009 and 2023, with most studies conducted in 2022 (*n* = 4). Female samples ranged from 5 participants [58] to 331 participants [76], with a total of 1337 female participants ranging from 10 to 19 years of age (one study did not report total female specific participant information [77]). Most studies contained samples from Europe (*n* = 3), Australia (*n* = 3), and the United States (*n* = 3), followed by Africa (*n* = 2), and Asia (*n* = 1). Regarding research design, eight were non-randomised studies (e.g., non-randomised controlled trial, pre-post, longitudinal cohort), two were randomised controlled trials (RCTs), and two were mixed methods. Of these, six studies included a follow-up period post-intervention ranging from one week to 12 months (*M* = 17 weeks, *SD* = 16.90). The interventions were mostly delivered within the school setting (*n* = 10) or the sport setting (*n* = 2), ranging from one to eight sessions (three studies did not specify number of sessions). Of these, 11 were face-to-face and one was online.

Mental health literacy overall (*n* = 5) and stigmatising attitudes (*n* = 5) were the most assessed outcomes, followed by knowledge (*n* = 4), help-seeking provision (*n* = 3), and social distancing (*n* = 1). A total of five studies addressed intervention implementation outcomes. Further details of included studies are presented in Table 2.

**Table 2:** Summary of included studies

Author & Year	Participants	Study design	Intervention details	Intervention setting	Follow up	Outcomes assessed; measures	Control description	Main findings
Bella-Awusah et al. 2014^a^	*N* = 80 *N*_I_ = 41 *N*_C_ = 39 *M*_age_ = unclear (range 10 – 18)	NRCT	No intervention name Face-to-face; 1 session, 180 min	School	6 months	Knowledge, attitudes & social distance; modified UK pinfold questionnaire	Untreated control No intervention	Knowledge significantly increased immediately and 6 months post-intervention (p < .001) No significant changes in attitudes or social distance
Hassen et al. 2022^a^	*N* = 67 *N*_I_ = 34 *N*_C_ = 33 *M*_age_ = unclear (range 15 – 19)	NRCT	Mental health Curriculum Online; 8 sessions across 6 weeks	School	N/A	MHL; MHLq implementation outcomes	Active control online; 8 sessions across 6 weeks	A significant change in MHL in the intervention group compared to the control group (*p* = .00) Females were satisfied and reported that the intervention was mostly acceptable, feasible, and appropriate
Morgado et al. 2022	*N* = 11 *M*_age_ = 14.09 (no age range)	MM exploratory	ProLiSMental Psychoeducational Intervention Face-to-face; 4 weekly sessions, 90 min each	School	N/A	Acceptability & feasibility; observation grids & satisfaction questionnaire	N/A	There were high levels of participation, acceptability, feasibility, and satisfaction from students
Naylor et al. 2009	*N* = 66 *M*_age_ = unclear (range 14 – 15)	Pre-post (evaluation data only)	No intervention name Face-to-face; 6 weekly sessions; 50 min each	School	N/A	Evaluation; 2 items for each topic made for study	Waitlist control	Female participants viewed the intervention highly, with important lessons being learned about suicide/self-harm
Panza et al. 2022	*N* = 5 *M*_age_ = unclear (range 13 – 16)	MM (qualitative data only)	Team talk Face-to-face; 1 session, duration not reported	Sport	N/A	Acceptability of implementation	N/A	Feedback from participants revealed that the intervention was feasible, acceptable and in-demand. Participants also described check-in meetings and pre-season intervention as improvements
Patafio et al. 2021	*N* = 138 (range 12 – 15)	NRCT	Read the play Face-to-face; 1 session, 60 min	Sport	2 weeks – 1 month	MHL; MHLS Help-seeking intentions; GHSQ Help-seeking behaviour; AHSQ	Pseudo-waitlist control	MHL remained consistent from pre to post intervention The intervention did not influence help-seeking intentions and behaviour MHL significantly decreased for females in the control condition
Perry et al. 2014^a^	*N* = total unclear *N*_I_ = total unclear *N*_C_ = total unclear *M*_age_ = 14.75 (range 13 – 16)	C-RCT	HeadStrong Face-to-face; sessions not reported, 10 h total	School	6 months	MHL; modified D-Lit stigma; DSS-personal attitudes; IASMHS	Treatment as usual PDHPE Class	Large effects on MHL were found post-intervention, with moderate effects maintained 6 months Weak effects on stigma, and attitudes were found post-intervention, with stigma having a small effect at 6 months
Pinto-Foltza et al. 2011^a^	*N* = 144 *N*_I_ = 89 *N*_C_ = 55 *M*_age_ = 15 (range 13 – 17)	C-RCT	In Our own voice Face-to-face; 1 session, 60 min	School	T3: 1 month T4: 2 months	MHL; in our own voice knowledge measure stigma; subscale of the revised attribution questionnaire feasibility; 3 items made for study acceptability; questionnaire based on Fischer’s narrative Paradigm theory & 1 item made for study	Untreated control No intervention	MHL significantly improved only at 4- and 8-weeks post-intervention The intervention did not have an intermediate effect on stigma The intervention was feasible to administer and retain participants. Enrolling participants is not feasible. Participants found the intervention highly acceptable and believable
Russell et al. 2023	*N* = 331 *M*_age_ = unclear (range 11 – 14)	Pre-post	Peer education Project Face-to-face; 5 classroom sessions, duration not reported	School	1 – 12 weeks	Knowledge; 12 items for study Help-seeking intentions; Adapted-GHSQ Help-seeking confidence; 12 items for study Perceived peer support; SBS	N/A	Intervention had a small effect on help-seeking intentions, knowledge and perceived peer support and a weak effect on help-seeking confidence
Wei et al. 2022	*N* = 105 *M*_age_ = unclear (Grade 12)	LC	Know before you go Face-to-Face; sessions not reported, 9 – 10 h total	School	1 month	Knowledge; 20 items for study	N/A	There was a small effect on knowledge 1 month after exposure of the program
Wei et al. 2023	*N* = 170 *M*_age_ = unclear (range 14 – 15)	LC	The Guide Face-to-Face; 1 session, duration not reported	School	12 months	Stigma; adapted youth opinion survey	N/A	Attitudes improved from pre-test to 12 month follow-up after the intervention
Zare et al. 2021^a^	*N* = 220 *N*_I_ = 110 *M*_age_^I^ = 13.75 *N*_C_ = 110 *M*_age_^C^ = 13.84 (range 13 – 15)	NRCT	Mental health & high school curriculum guide Face-to-face; 6 sessions, 60 – 90 min each	School	N/A	MHL; Adapted mental health literacy questionnaire with subscales first aid skills and help-seeking, knowledge/stereotypes, and self-help strategy	Treatment as usual Usual school program	MHL significantly increased post-intervention compared to control (p < .01) First aid skills and help-seeking, knowledge/stereotypes, and self-help strategy significantly increased post-intervention compared to control (p < .01)

*NRCT* non-randomised controlled trial (e.g., quasi-experimental), *C-RCT* cluster-randomised controlled trial, *MM* mixed methods, *LC* longitudinal cohort, *MHL* mental health literacy

^a^Studies included in meta-analysis. Details reported are based on available female data only

### Study quality

A total of five studies were assessed using the JBL checklist for quasi-experimental studies, two studies using the JBL checklist for randomised controlled trials, two studies using the JBL checklist for cohort studies, two studies using the MMAT, and one study using the NHLBI tool for before-after (pre-post) studies with no control group. Overall, all studies met at least five criteria from their respective tools, with eight studies receiving an unclear or cannot determine comment on at least one criterion. The results of the study quality analysis are provided in Supplementary File 2.

### Summary of included interventions

The included studies implemented a variety of interventions, for example, Mental Health Curriculum [78, 79], ProLiSMental Psychoeducational Intervention [80], Read the Play [81], In Our Own Voice [82], The Guide [83], Know Before You Go [84], Peer Education Project [76], HeadStrong Program [77], and Team Talk [58]. These interventions all shared a general aim to enhance mental health awareness, reduce stigma, promote help-seeking behaviours, and equip participants with skills to support themselves and peers’ mental wellbeing (*n* = 10). Some interventions broadened their scope to also include life skills such as resilience, academic planning, and career development (e.g., [77, 84]). While many interventions focused on general mental health (e.g., [76, 78, 83]), others addressed specific conditions like anxiety, mood disorders, personality disorders, eating disorders, sexual health, suicide and self-harm, and bullying through targeted modules (e.g., [80, 81]). Skill development was also a common component across interventions, with sessions on recognising symptoms, understanding risks, and building self-help strategies such as resilience and stress management (e.g., [77, 82]). However, only a few interventions explored other cultural and social risk factors and social identities, examining media portrayals of mental illness and relationships (e.g., [58, 84]). Engagement methods across all interventions included interactive approaches like role-plays, group discussions, and games, with some interventions opting for traditional formats like lectures and independent study (e.g., [79]).

### Meta-analyses

**Fig. 2:**
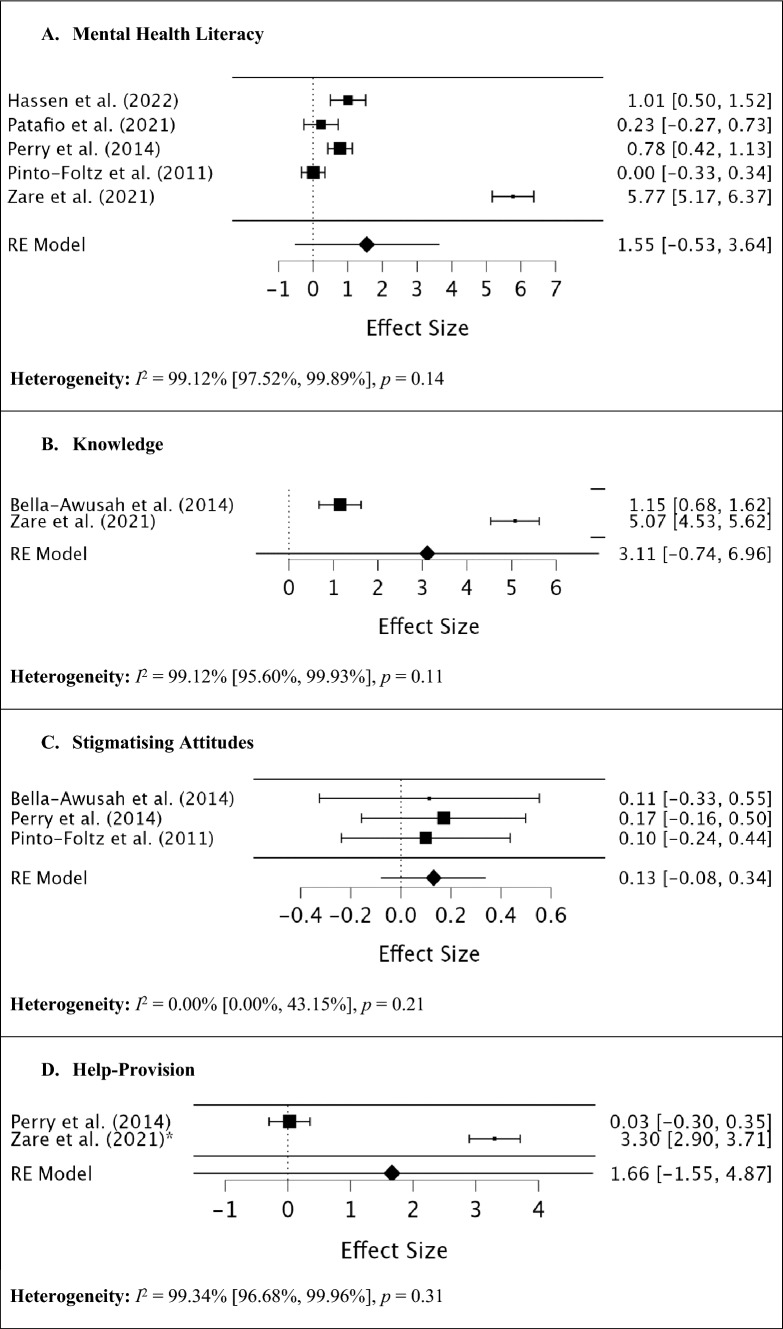
Meta-analyses for each of the mental health literacy outcome domains at post-intervention *Denotes that *d* was combined from several effect sizes in a single study

**Fig. 3:**
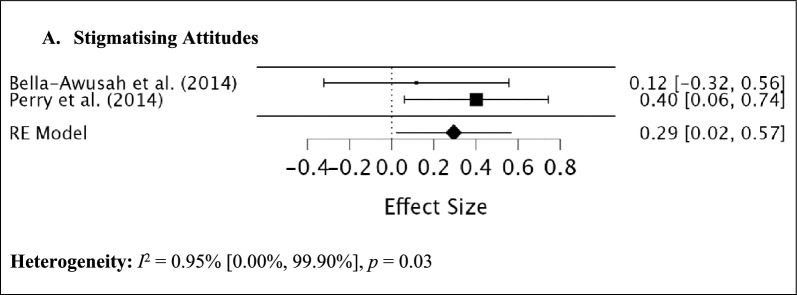
Meta-analyses for the mental health literacy outcome domains at 6 months post-intervention

Although meta-analyses were planned for each of the mental health literacy outcomes at post-intervention and > 6-months post-intervention, this could not be conducted for outcomes that had fewer than two effect sizes. As a result, the evaluation of mental health literacy, knowledge, stigmatising attitudes, and helping-seeking provision were all assessed post-intervention, and only stigmatising attitudes was assessed > 6 months post-intervention. The results of the meta-analyses on mental health literacy outcomes are displayed in Figs. 2 and 3.

### Evaluation of mental health literacy

A total of five studies (three N-RCTs and two RCTs) that included a measure of mental health literacy at post-intervention were included in the meta-analysis, with a total number of 702 female adolescents (13 – 19 years). The test of residual heterogeneity was high, *I*^*2*^ = 99.12%, *Q* (4) = 287.21, *p* > 0.001. The random-effects meta-analysis identified no significant differences in mental health literacy post-intervention (*d* = 1.55, 95% CI = -0.53, 3.64, *p* = 0.14).

### Evaluation of mental health knowledge

A total of two studies (two N-RCTs) that included a measure of mental health knowledge at post-intervention were included in the meta-analysis, with a total number of 300 female participants (10 – 18 years). The test of residual heterogeneity was high, *I*^*2*^ = 99.12%, *Q* (1) = 114.22, *p* > 0.001. The random-effects meta-analysis identified no significant differences in mental health knowledge at post-intervention (*d* = 3.11, 95% CI = -0.74, 6.96, *p* = 0.11).

### Evaluation of stigmatising attitudes

A total of three studies (one N-RCT and two RCTs) that included a measure of stigmatising attitudes at post-intervention were included in the meta-analysis, with a total number of 369 female participants (10 – 18 years). The test of residual heterogeneity was low, *I*^*2*^ = 0.00%, *Q* (2) = 0.10, *p* = 0.95. The random-effects meta-analysis identified no significant differences in stigmatising attitudes at post-intervention (*d* = 0.13, 95% CI = -0.08, 0.34, *p* = 0.21). At > 6-month follow-up, two studies (one N-RCT and one RCT) were included in the meta-analysis, with a total number of 215 female adolescents (10 – 18 years). The test of residual heterogeneity was also low, *I*^*2*^ = 0.95%, *Q* (1) = 1.01, *p* = 0.31. The random-effects meta-analysis identified a significant small difference in stigmatising attitudes > 6-months following the intervention (*d* = 0.29, 95% CI = 0.02, 0.57, *p* = 0.03).

### Evaluation of help-seeking provision

A total of two studies (one N-RCT and one RCT) that included a measure of help provision at post-intervention were included in the meta-analysis, with a total number of 364 female participants (13 – 16 years). The test of residual heterogeneity was high, *I*^*2*^ = 99.34%, *Q* (1) = 151.53, *p* > 0.001. The random-effects meta-analysis identified no significant differences in help provision at post-intervention (*d* = 1.66, 95% CI = -1.55, 4.87, *p* = 0.31).

### RE-AIM criteria

A summary of the RE-AIM results by each criterion can be found in Supplementary File 3.

### Reach

Overall, reach was poorly reported by all the studies in this review. All 12 studies outlined their target population identification and recruitment methods. However, only three studies detailed an exclusion criterion [78, 79, 82], with one study explicitly stating having no exclusion criteria [77]. Participant exclusion criteria across these studies included adolescents below the age of 15 years, those with a sibling in the adjacent grade, male adolescents, missing two or more education sessions, and leaving the research setting (e.g., changing schools). Additionally, eight studies reported participation percentages based on those approached, however, only two of these studies reported percentages for female participants specifically, with 22% of female students participating [80, 82]. The remaining six studies did not specify percentages for female participants exclusively [58, 76, 78, 81, 83, 85]. No studies measured characteristics of participants compared to non-participants or target population, as well as the use of qualitative methods to understand reach and recruitment.

### Effectiveness

Effectiveness was the best reported outcome included in this review. All studies included strong reporting of changes in primary outcomes (e.g., mental health literacy [78]), with three of these studies reporting on broader outcomes (e.g., mental wellbeing, [76]). Participants reported high levels of satisfaction and acceptability of the intervention [78], and that they were most satisfied with the trainers, adequacy of the activities, and the relevance of the content [80]. Participants also reported learning a lot about self-harm and suicide, followed by depression, intellectual disabilities, bullying, eating disorders, and stress. Of these, self-harm and suicide, bullying and eating disorders were most important to females [85]. A total of three studies reported short-term attrition rates for female participants, which averaged to be approximately 23.7% (8% to 37%). Of these, only one study compared characteristics of female adolescents who dropped out and those who did not, which found no systematic differences [82]. Intention-to-treat (*n* = 3) and robustness (*n* = 2) analyses across groups was poorly conducted across studies. A total of two studies used qualitative methods to measure intervention effectiveness. Both studies had high rates of acceptability as participants reported that they enjoyed the intervention and storytelling components, absorbed the general goal of the intervention, strengthened their bonds with their teammates and team identity, and improved their willingness to discuss topics around mental health [58, 82].

### Adoption

In general, reporting on adoption at the setting and staff level was inadequate across all reviewed studies. At the setting level, five studies disclosed details on the percentage of approached settings that participated, with 25% of schools [76, 77, 83, 84] and 1% of sporting clubs participating [58]. Only one study specified a setting exclusion criterion, being public schools [77]. Moreover, only one study provided characteristics of participating settings compared to non-participating ones, identifying lack of time as a key factor [58]. Panza et al. [58] was also the only study that used qualitative methods to understand adoption and suggested that in the sporting context, presenting interventions early in the season and having regular check-in meetings will enhance the adoption of the intervention. At the staff level (excluding [78], due to the online nature of the intervention), only Pinto-Foltz et al. [82] specified a staff exclusion criterion, restricting staff participation to ages 18 and above. No studies reported on staff percentage participation, characteristics between participating staff and non-participation staff, and qualitative methods to understand staff participation.

### Implementation

Implementation was another poorly reported RE-AIM construct. Pinto-Foltz et al. [82] and Panza et al. [58] found that while study enrolment feasibility was not well supported, participant retention was relatively high once enrolled. Participants themselves also reported that the intervention was feasible and appropriate [78]. Only four studies mentioned changes made to the intervention to facilitate delivery, such as adjusting the order of topics [77], targeting different year groups [76], and modifying intervention activities [83, 84]. A total of four studies based their intervention on a guiding theory, such as the theory of change [78], theory of planned behaviour [80], social identity theory [58], and Fischer’s narrative paradigm theory [82].

Fidelity, or the adherence to the intended intervention, was only assessed in one study using audiotapes [82]. While Panza et al. [58] did not report on specific percentages, they used researcher logs to determine general experience of delivery, revealing time availability and coach involvement as challenges to intervention implementation. Only one study reported low implementation costs due to the online nature of the intervention [78]. No studies commented on the consistency of implementation across settings. One study used qualitative methods to measure implementation [80]. The study found that students mostly favoured the contents (e.g., emotions, anxiety, self-help strategies and sources of help), pedagogical methods (e.g., reflective questions, demonstrative examples), and activities of the program (e.g., role-play, working groups), while specific subjects (e.g., mental illness’ myths) and evaluation strategies (e.g., questionnaires) were less popular. They suggested reducing topics on mental illness’ myths and including more relaxation and educational training, role-plays, working groups, and anxiety assessment questionnaires [80].

### Maintenance

Maintenance at the individual and setting level was also poorly reported on by all the studies included for review. Beyond immediate post-intervention measurements, only three studies assessed primary outcomes at 6 months [77, 86] and 12 months [83], with one study also assessing broader outcomes at 6 months [77]. These three studies addressed participant loss during follow-up to some degree. For example, Perry et al. [77] conducted a missing data analysis and Wei et al. [83] attributed attrition to student and teacher turnover. No studies employed qualitative methods to explore long-term impacts, and none reported on setting-level maintenance across all criteria.

## Discussion

The aim of the current review was to assess and evaluate mental health literacy interventions for female adolescents. No interventions in the current study were specifically catered to female adolescents. With respect to mental health literacy outcomes, there were no significant effects immediately post-intervention, however, stigmatising attitudes was significant > 6-months post-intervention. Due to the limited number of studies included for review, it was not feasible to evaluate the factors moderating these effects, despite the heterogeneity observed across most outcomes. The RE-AIM framework was also used to systematically examine these interventions, with reach, adoption, and maintenance being the most poorly reported across all studies. Together, these results highlight that there is substantial room to improve the effectiveness and implementation of mental health literacy interventions delivered for female adolescents.

### Evaluation of mental health literacy outcomes

Overall, there were no significant effects of mental health literacy interventions on outcomes within the categories of mental health literacy, knowledge, stigmatising attitudes, and help-provision immediately post-intervention. Similar findings have been reported in previous reviews regarding stigmatising attitudes, and help-provision [44, 46]. However, these results contrast with other reviews on mental health literacy overall [45] and knowledge [44, 46, 87], which reported significant improvements post-intervention. Importantly, these reviews were inclusive of male and female adolescents, presenting challenges in making comparisons to the current study. One possible explanation for these non-significant results is the lack of female-specific mental health literacy interventions. Although female adolescents typically exhibit higher levels of mental health literacy compared to males [38], the current interventions were designed for adolescents as a homogenous group. Consequently, they may not address the unique developmental, social, and psychological needs of female adolescents, thus limiting their relevance and effectiveness for this group. A review by Herrmann et al. [49] highlights the increased effectiveness of gender-specific mental health prevention programs for young people. These approaches are particularly evident in addressing specific conditions such as eating disorders (e.g., [88]) and substance use problems (e.g., [89]) for girls, and more recently in initiatives for women in rugby [90]. However, gender-specific interventions for broader mental health literacy are notably absent for female adolescents. Conversely, gender-specific strategies for men and boys mental health literacy are more prevalent and widely accepted, with interventions in both sport (e.g., Ahead of the Game, [91–94]; Talk Today, [95]) and community settings (e.g., Reach Out Central, [96]) [97, 98]. This further underscores the absence of female-specific mental health literacy interventions, highlighting a lack in resources and support tailored to female adolescents.

To develop effective female-specific mental health interventions, it is essential to consider topics and engagement methods that resonate with female adolescents, distinguishing them from gender-neutral and male-specific approaches. Previous research [49, 80, 85] and findings from this review highlight that topics such as eating disorders, bullying, and suicide/self-harm are particularly relevant for female adolescents. Additionally, female adolescents consistently highlight the importance of interactive engagement methods like role-plays and group discussions [80], reflecting their preference for informal, emotionally supportive relationships with peers and friends [99]. However, currently available interventions are either: (1) gender-neutral and broadly address mental health literacy (e.g., The Guide [83], Peer Education Project [76]), or (2) male-specific and address mental health literacy within the male context (e.g., signs and symptoms of anxiety and depression for males [91–94] and use individualised computer-based game formats [96]). Given the topic and activity preferences of female adolescents, it may be that incorporating additional topics like eating disorders and suicide/self-harm and diversifying engagement activities are essential for an intervention targeting the needs of females, going above what is currently available. Such targeted efforts are fundamental in promoting positive well-being and mental health literacy among female adolescents [100]. In future, researchers are encouraged to further explore what female adolescent’s want and need in a gender-specific mental health literacy intervention, building an evidence base that supports effective, high impact mental health promotion and improves outcomes for this high-risk group.

In addition, the diverse operationalisation and measurement approaches for each mental health literacy construct may have influenced the results [101]. Most articles predominately relied on Jorm et al.’s [28] definition, which focuses on mental ill-health [77, 78, 81, 82], without considering broader interpretations like that of Kutcher et al. [29] which acknowledges mental health beyond the absence of illness. As well as this, measurement inconsistencies were evident such that some studies included mental health literacy in its broader sense [78, 81], while others only addressed specific constructs (e.g., [76, 86]). This lack of measurement consistency may result in construct stretching and heterogeneous measurement [102]. Reviews on the psychometric properties of mental health literacy support the need for a shared definition to enhance measurement consistency [103–107]. This approach can inform future strategies to improve mental health literacy at both individual and community levels [107]. Importantly, there was also evidence of heterogeneity across three meta-analyses, indicating variability between studies. However, given the small number of studies that measured mental health literacy, knowledge, and help-seeking provision, it was not possible to assess the factors moderating these effects.

Consistent with a previous review [108], stigmatising attitudes was the only outcome measured at > 6-months post-intervention, with results revealing a small favourable effect of mental health literacy interventions on stigmatising attitudes. This suggests that over time, stigmatising attitudes may have reduced as a result of the intervention. One potential reason for this is that attitudinal change typically evolves gradually, indicating that changes can only be seen over the long-term. However, the two included studies [77, 86] were not significantly heterogenous, indicating that the underlying contextual elements influencing these effects remain largely unknown. Therefore, while the evidence suggests a potential benefit of mental health literacy interventions in reducing stigmatising attitudes over time, the findings should be taken with caution.

### RE-AIM outcomes

This review also systematically examined the reporting of the RE-AIM framework of mental health literacy interventions for female adolescents. Generally, there was limited reporting on all components of the RE-AIM framework at both the individual and setting levels. While it is also acknowledged that some of these components may not have been the aim of the studies, reach, adoption, and maintenance were the most poorly reported across all studies. This is consistent with other reviews using the RE-AIM framework for health literacy and behavioural interventions [109–111]. Importantly, these conclusions do not reflect the overall studies or the uptake of the intervention itself; they only reflect the information currently available for female adolescents.

While many studies outlined their target population and recruitment methods, there is a notable gap in reporting participation rates and sample characteristics specified by gender. That is, most studies did not differentiate these factors between male and female adolescents. This lack of reporting may be attributed to the inherent design of scale-up interventions, where the aim is to maximise the exposure of participations within the given context to the intervention [110]. Similarly, adoption was inadequately documented with few studies examining the details of setting characteristics and staff demographics among intervention deliverers. This includes participation percentages, disparities between participants and non-participants, and qualitative insights. This lack of data on participation, staff, and setting factors, highlights the need for future research to robustly investigate recruitment and training strategies aimed at optimising intervention uptake [109].

Individual reporting of intervention effectiveness and implementation varied across studies, with most showing favourable mental health literacy outcomes immediately post-intervention. This suggests that while the meta-analyses may not be significant, there is potential for meaningful impact through well-designed, and adequately implemented interventions. However, limitations such as lack of attrition reporting and intention-to-treat analyses may lead to potential overestimation of findings. Furthermore, there was limited evidence regarding the individual and setting level maintenance, suggesting that the longer-term impacts of mental health literacy interventions for females is not adequately described or evaluated. This emphasises a gap in understanding the sustained impact of mental health literacy interventions, highlighting the need for more robust longitudinal studies to assess effectiveness over time [108].

Despite this, participants generally expressed high satisfaction with intervention components such as trainers [80], activities [82], content (e.g., self-harm and suicide; [85]) and found them engaging, feasible and appropriate [58, 78]. However, there was inconsistency in the depth of theoretical underpinnings provided for the interventions, with only four studies offering clear theoretical frameworks [58, 78, 80, 82]. Additionally, factors influencing the translation of mental health literacy interventions such as adaptations, adherence, and cost delivery was inadequately reported, which is consistent with other RE-AIM reviews on health literacy [111–113]. Without this knowledge, it is difficult to gauge whether interventions are meeting the minimum requirements for improving mental health literacy outcomes, raising questions around feasibility and sustainability for female adolescents. Overall, this lack of comprehensive reporting creates significant gaps in understanding how to effectively translate and scale mental health literacy interventions. This highlights that more studies are needed that focus on the conditions in which the intervention is carried out. Therefore, a hybrid approach integrating effectiveness and implementation designs should be considered to bridge this gap and provide greater transparency in informing practice changes and implementations [114].

### Theoretical and practical implications

The current systematic review and meta-analysis contributes to the theoretical understanding of mental health literacy by emphasising the need for continued exploration and application of its constructs, particularly in the context of female adolescents. This review highlights that most existing studies have not utilised the full framework of mental health literacy, often focusing on only one or two constructs rather than the broader model. This gap underscores the necessity for future research to address all dimensions of mental health literacy to provide a more comprehensive understanding and accurately capture the mental health literacy needs of female adolescents. Regarding practical implications, this review highlights a significant gap in female-specific mental health literacy interventions, revealing a lack of tailored resources and support for this high-risk group. While female adolescents report more favourable help-seeking intentions than male adolescents when equipped with the appropriate skills and resources [115, 116], the current review establishes existing interventions are often failing to meet their needs. This emphasises the urgent need to develop effective, inclusive, and gender-responsive mental health promotion strategies to ensure equitable access to support. Additionally, by identifying what works and resonates with female participants in current programs, this study offers a blueprint for the development of targeted interventions to meet the specific needs of this vulnerable group. It is also important to consider that additional avenues may need to be explored beyond mental health literacy to enhance mental health outcomes for female adolescents, such as treatment-based approaches and policy changes that address underlying mental health risk factors and social determinants of mental health. However, given the demonstrated effectiveness of male-specific interventions [91–94], and the notable absence of female-specific ones, prioritising the exploration and development of female-specific interventions that apply the mental health literacy framework in its entirety is a crucial initial step.

### Strengths and limitations

This study addresses a previously unexplored area by systematically reviewing and meta-analysing mental health literacy interventions for female adolescents. Additionally, the comprehensive search strategy used, which included six electronic databases (PsycInfo, PsycArticles, CINAHL Plus, MEDLINE, Sport Discus, and Scopus) and a systematic approach to find all available studies across the areas of health, psychology, and medicine are both key strengths of this study. However, there are several limitations related to this review and the broader mental health literacy intervention literature that are important to acknowledge. Relevant to this review, the range of conceptualisations and definitions of mental health literacy outcomes may have reduced the overall intervention effects. Of the studies measuring female mental health literacy, few studies measured all components of mental health literacy (i.e., five out of nine studies measured only some components). Ideally, all components of mental health literacy should be measured, so future research should ensure comprehensive measurement of all relevant components when assessing mental health literacy. Additionally, although a protocol for this review was developed and published on Prospero, some deviations were made to enhance the review. For example, an additional study quality analysis was included to ensure accurate assessment. While this was clearly communicated, it is believed to have improved the review’s quality.

Relevant to the mental health literacy intervention literature more generally, no interventions in the current study specifically catered to female adolescents. This may have reduced intervention effects for this group as the current interventions were developed for the general adolescent population. Future research should identify key factors influencing mental health promotion for female adolescents to develop and implement more effective, gender-specific interventions. In addition to this, there was a substantial amount of missing data for female adolescents. Many studies deemed appropriate during the full-text screening lacked the necessary demographic data. Despite obtaining authors contact details and sending an email request, the response rate was low, with only three out of 29 (10.34% response rate) authors providing the requested information. As a result, many studies that may have made a meaningful contribution to this review were excluded for not differentiating data between male and female adolescents. Therefore, the findings of this review should be interpreted with caution as it not an accurate representation of all the literature on female adolescents’ mental health literacy. Future research is encouraged to follow guidelines such as the Sex and Gender Equity in Research (SAGER) Guidelines Checklist to ensure more systematic reporting of sex and gender [117]. Further, there were few studies in this review that included randomisation designs (e.g., quasi-experimental, pre-post design), limiting the confidence in the results due to potential confounding variables. This highlights a significant opportunity for future research to strengthen study designs (e.g., randomised controlled trials) in this area.

## Conclusion

In conclusion, this review found mixed evidence regarding the impact of mental health literacy interventions for female adolescents. Although these interventions appear to be feasible, acceptable, and interesting, their pooled effects on mental health literacy were not effective. The lack of female-specific interventions, contrasted with the available male-specific ones, highlights a significant deficiency in tailored mental health prevention and promotion interventions for this group. As such, there is a clear need to understand and target female adolescents’ specific mental health literacy needs, as well as enhance the quality of these interventions, including stronger study designs, conceptualisation and measurement and improved implementation protocols. This knowledge will be crucial for the effective development, distribution, and implementation of interventions that meet the mental health literacy needs of female adolescents.

## Supplementary Information

Below is the link to the electronic supplementary material.

Supplementary file1 (DOCX 201 KB)

Supplementary file2 (DOCX 33 KB)

Supplementary file3 (DOCX 28 KB)

## Data Availability

No datasets were generated or analysed during the current study.
