# Supplementary material for: Mental health literacy interventions for female adolescents: a systematic review and meta-analysis
Source: Eur Child Adolesc Psychiatry. 2025 Jan 22;34(6):1749–67. doi: 10.1007/s00787-025-02648-2 (PMC12198331; doi:10.1007/s00787-025-02648-2)
Supplement: Supplementary file 1 — Supplementary file1 (DOCX 201 KB) [file 787_2025_2648_MOESM1_ESM.docx]

**Mental Health Literacy Interventions for Female Adolescents: A Systematic Review and Meta-Analysis**

European Child & Adolescent Psychiatry

Emily R Arnold*^1^, Caitlin Liddelow^1^, Angie S X Lim^1^, Stewart A Vella^1^,

^1^ Global Alliance for Mental Health and Sport (GAMeS), School of Psychology, Faculty of the Arts, Social Sciences and Humanities, University of Wollongong, Northfields Avenue, Wollongong, NSW 2522

***Corresponding author:** Emily R Arnold - Global Alliance for Mental Health and Sport (GAMeS), School of Psychology, Faculty of the Arts, Social Sciences and Humanities, University of Wollongong, Northfields Avenue, Wollongong, NSW 2522. [era391@uowmail.edu.au](mailto:era391@uowmail.edu.au).

**Post-Intervention**

| **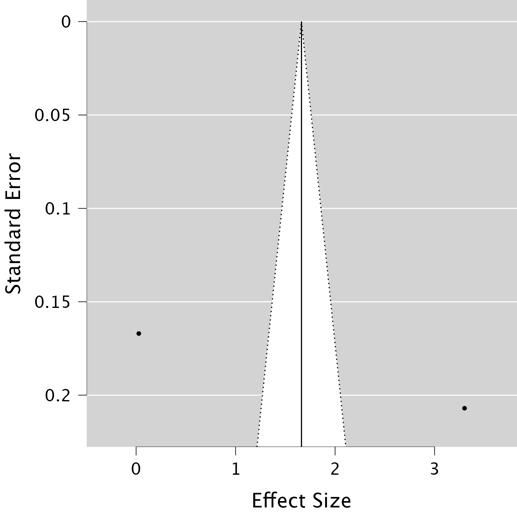Help-Seeking Provision** |
| --- |
| **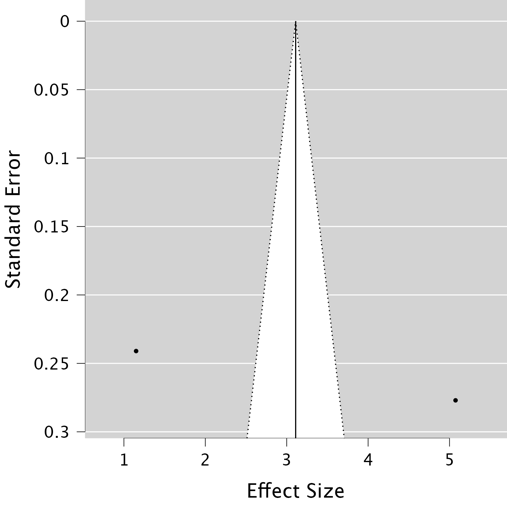Knowledge** |
| **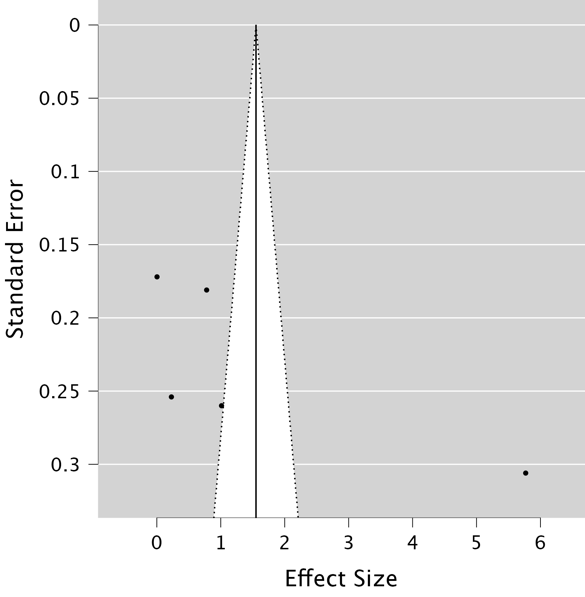Mental Health Literacy** |
| **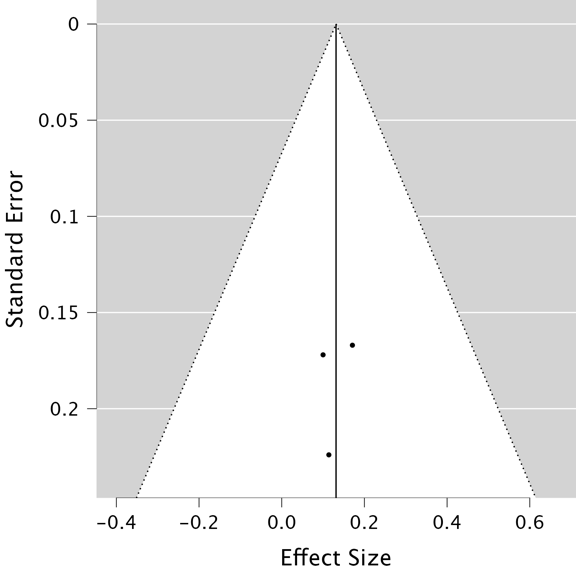Stigmatising Attitudes** |

**>6-months Post-Intervention**

| **Mental Health Literacy**  **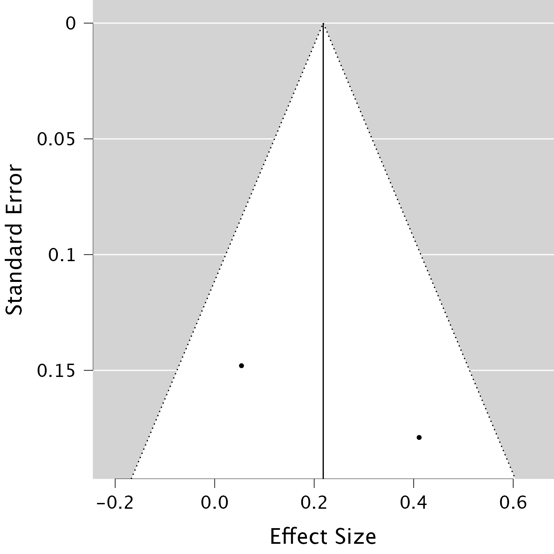** |
| --- |
| **Stigmatising Attitudes**  **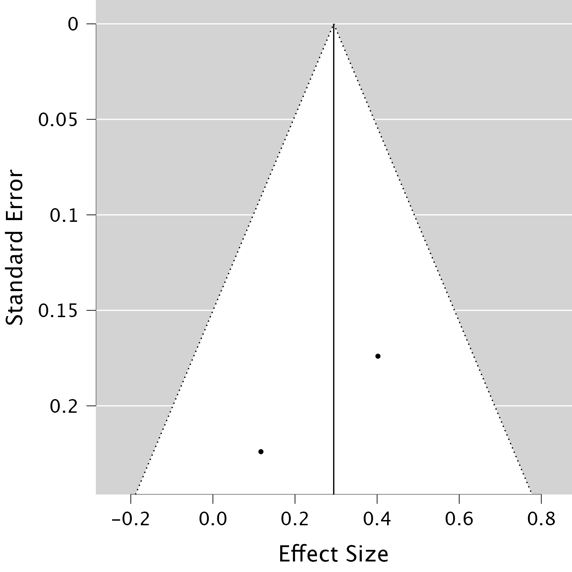** |
