# Supplementary material for: Mental health literacy interventions for female adolescents: a systematic review and meta-analysis
Source: Eur Child Adolesc Psychiatry. 2025 Jan 22;34(6):1749–67. doi: 10.1007/s00787-025-02648-2 (PMC12198331; doi:10.1007/s00787-025-02648-2)
Supplement: Supplementary file 2 — Supplementary file2 (DOCX 33 KB) [file 787_2025_2648_MOESM2_ESM.docx]

**Mental Health Literacy Interventions for Female Adolescents: A Systematic Review and Meta-Analysis**

European Child & Adolescent Psychiatry

Emily R Arnold*^1^, Caitlin Liddelow^1^, Angie S X Lim^1^, Stewart A Vella^1^,

^1^ Global Alliance for Mental Health and Sport (GAMeS), School of Psychology, Faculty of the Arts, Social Sciences and Humanities, University of Wollongong, Northfields Avenue, Wollongong, NSW 2522

***Corresponding author:** Emily R Arnold - Global Alliance for Mental Health and Sport (GAMeS), School of Psychology, Faculty of the Arts, Social Sciences and Humanities, University of Wollongong, Northfields Avenue, Wollongong, NSW 2522. [era391@uowmail.edu.au](mailto:era391@uowmail.edu.au).

| **Criteria** | **Non-Randomised Controlled Trial** | | | | | | **Mixed Method** | | **Randomised Controlled Trial** | | | **Cohort Study** | | **Before-After Study with No Control Group** | |
| --- | --- | --- | --- | --- | --- | --- | --- | --- | --- | --- | --- | --- | --- | --- | --- |
|  | Bella-Awusah et al.  2014 | | Hassen et al.  2022 | Naylor et al.  2009 | Patafio et al.  2021 | Zare et al. 2021 | Moco Morgado et al.  2022 | Panza et al.  2022 | Perry et al.  2014 | Pinto-Foltz et al.  2011 | Wei et al.  2022 | | Wei et al.  2023 | | Russell et al.  2023 |
| **Non-Randomised Controlled Trials** | | |  | | | | | | | | | | | | |
| Is it clear in the study what is the ‘cause’ and what is the ‘effect’ | Y | | Y | Y | Y | Y |  |  |  |  |  | |  | |  |
| Were the participants included in any comparisons similar? | Y | | Y | Y | UC | Y |  |  |  |  |  | |  | |  |
| Were the participants included in any comparisons receiving similar treatment/care, other than the exposure or intervention of interest? | UC | | UC | UC | Y | UC |  |  |  |  |  | |  | |  |
| Was there a control group? | Y | | Y | Y | Y | Y |  |  |  |  |  | |  | |  |
| Were there multiple measurements of the outcome both pre and post the intervention/exposure? | Y | | Y | Y | Y | Y |  |  |  |  |  | |  | |  |
| Was follow up complete and if not, were differences between groups in terms of their follow up adequately described and analyzed? | Y | | UC | UC | Y | UC |  |  |  |  |  | |  | |  |
| Were the outcomes of participants included in any comparisons measured in the same way? | Y | | Y | Y | Y | Y |  |  |  |  |  | |  | |  |
| Were outcomes measured in a reliable way? | Y | | N | Y | Y | Y |  |  |  |  |  | |  | |  |
| Was appropriate statistical analysis  used? | N | | Y | Y | Y | Y |  |  |  |  |  | |  | |  |
| **Mixed Methods** |  | |  |  |  |  |  |  |  |  |  | |  | |  |
| Are there clear research questions? |  | |  |  |  |  | Y | Y |  |  |  | |  | |  |
| Do the collected data allow to address the research questions? |  | |  |  |  |  | Y | Y |  |  |  | |  | |  |
| Is there an adequate rationale for using a mixed methods design to address the research question? |  | |  |  |  |  | Y | Y |  |  |  | |  | |  |
| Are the different components of the study effectively integrated to answer the research question? |  | |  |  |  |  | Y | Y |  |  |  | |  | |  |
| Are the outputs of the integration of qualitative and quantitative components adequately interpreted? |  | |  |  |  |  | Y | Y |  |  |  | |  | |  |
| Are divergences and inconsistencies between quantitative and qualitative results adequately addressed? |  | |  |  |  |  | Y | Y |  |  |  | |  | |  |
| Do the different components of the study  adhere to the quality criteria of each tradition of the methods involved? |  | |  |  |  |  | Y | Y |  |  |  | |  | |  |
| **Randomised Controlled Trials** |  | |  |  |  |  |  |  |  |  |  | |  | |  |
| Was true randomization used for assignment of participants to treatment groups? |  | |  |  |  |  |  |  | Y | Y |  | |  | |  |
| Was allocation to treatment groups concealed? |  | |  |  |  |  |  |  | Y | N |  | |  | |  |
| Were treatment groups similar at the baseline? |  | |  |  |  |  |  |  | Y | Y |  | |  | |  |
| Were participants blind to treatment assignment? |  | |  |  |  |  |  |  | N | N |  | |  | |  |
| Were those delivering the treatment blind to treatment assignment? |  | |  |  |  |  |  |  | N | N |  | |  | |  |
| Were treatment groups treated identically other than the intervention of interest? |  | |  |  |  |  |  |  | Y | N |  | |  | |  |
| Were outcome assessors blind to treatment assignment? |  | |  |  |  |  |  |  | Y | N |  | |  | |  |
| Were outcomes measured |  | |  |  |  |  |  |  | Y | Y |  | |  | |  |
| in the same way for treatment groups? |  | |  |  |  |  |  |  |  |  |  | |  | |  |
| Were outcomes measured in a reliable way? |  | |  |  |  |  |  |  | Y | Y |  | |  | |  |
| Was follow up complete and if not, were differences between groups in terms of their follow up |  | |  |  |  |  |  |  | Y | Y |  | |  | |  |
| adequately described and analysed? |  | |  |  |  |  |  |  |  |  |  | |  | |  |
| Were participants analysed in the groups to which they were randomized? |  | |  |  |  |  |  |  | Y | N |  | |  | |  |
| Was appropriate statistical analysis used? |  | |  |  |  |  |  |  | Y | Y |  | |  | |  |
| Was the trial design appropriate and any deviations  from the standard RCT design accounted for in the conduct and analysis of the trial? |  | |  |  |  |  |  |  | Y | N |  | |  | |  |
| **Cohort Study** |  | |  |  |  |  |  |  |  |  |  | |  | |  |
| Were the two groups similar and recruited from the same population? |  | |  |  |  |  |  |  |  |  | N/A | | N/A | |  |
| Were the exposures measured similarly to assign people to both exposed and unexposed groups? |  | |  |  |  |  |  |  |  |  | N/A | | N/A | |  |
| Was the exposure measured in a valid and reliable way? |  | |  |  |  |  |  |  |  |  | Y | | Y | |  |
| Were confounding factors identified? |  | |  |  |  |  |  |  |  |  | N/A | | N/A | |  |
| Were strategies to deal with confounding factors stated? |  | |  |  |  |  |  |  |  |  | N/A | | N/A | |  |
| Were the groups/participants free of the outcome at the start of the study (or at the moment of exposure)? |  | |  |  |  |  |  |  |  |  | UC | | UC | |  |
| Were the outcomes measured in a valid and reliable way? |  | |  |  |  |  |  |  |  |  | Y | | Y | |  |
| Was the follow up time reported and sufficient to be long enough for outcomes to occur? |  | |  |  |  |  |  |  |  |  | Y | | Y | |  |
| Was follow up complete, and if not, were the reasons to loss to follow up described and explored? |  | |  |  |  |  |  |  |  |  | UC | | Y | |  |
| Were strategies to address incomplete follow up utilized? |  | |  |  |  |  |  |  |  |  | Y | | Y | |  |
| Was appropriate statistical analysis used? |  | |  |  |  |  |  |  |  |  | Y | | Y | |  |
| **Before-After Study with No Control Group** | |  |  |  |  |  |  |  |  |  |  | |  | |  |
| Was the study question or objective clearly stated? |  | |  |  |  |  |  |  |  |  |  | |  | | Y |
| Were eligibility/selection criteria for the study population prespecified and clearly described? |  | |  |  |  |  |  |  |  |  |  | |  | | N |
| Were the participants in the study representative of those who would be eligible for the |  | |  |  |  |  |  |  |  |  |  | |  | | CD |
| test/service/intervention in the general or clinical population of interest? |  | |  |  |  |  |  |  |  |  |  | |  | |  |
| Were all eligible participants that met the prespecified entry criteria enrolled? |  | |  |  |  |  |  |  |  |  |  | |  | | Y |
| Was the sample size sufficiently large to provide confidence in the findings? |  | |  |  |  |  |  |  |  |  |  | |  | | N/A |
| Was the test/service/intervention clearly described and delivered consistently across the study population? |  | |  |  |  |  |  |  |  |  |  | |  | | Y |
| Were the outcome measures prespecified, clearly defined, valid, reliable, and assessed consistently across all study participants? |  | |  |  |  |  |  |  |  |  |  | |  | | Y |
| Were the people assessing the outcomes blinded to the participants' exposures/interventions? |  | |  |  |  |  |  |  |  |  |  | |  | | N |
| Was the loss to follow-up after baseline 20% or less? Were those lost to follow-up accounted for in the analysis? |  | |  |  |  |  |  |  |  |  |  | |  | | Y |
| Did the statistical methods examine changes in outcome measures from before to after the intervention? Were statistical tests done that provided p values for the pre-to-post changes? |  | |  |  |  |  |  |  |  |  |  | |  | | Y |
| Were outcome measures of interest taken multiple times before the intervention and multiple times after the intervention (i.e., did they use an interrupted time-series design)? |  | |  |  |  |  |  |  |  |  |  | |  | | N |
| If the intervention was conducted at a group level  (e.g., a whole hospital, a community, etc.) did the statistical analysis take into account the use of individual-level data to determine effects at the group level? |  | |  |  |  |  |  |  |  |  |  | |  | | N/A |

*Note.* Y = Yes, N = No, UC = Unclear, N/A = Not applicable, CD = Cannot determine. Non-randomised controlled trials, Randomised controlled trials & Cohort study = Joanna Briggs Institute (JBI) Critical Appraisal Tools. Mixed methods = Mixed Methods Appraisal Tool (MMAT) Version 2018. Before-after study with no control group = National Heart, Lung, and Blood Institute (NHLBI) assessment tool Quality Assessment Tool for Observational Cohort and Cross-Sectional Studies.
