# Supplementary material for: Mental health literacy interventions for female adolescents: a systematic review and meta-analysis
Source: Eur Child Adolesc Psychiatry. 2025 Jan 22;34(6):1749–67. doi: 10.1007/s00787-025-02648-2 (PMC12198331; doi:10.1007/s00787-025-02648-2)
Supplement: Supplementary file 3 — Supplementary file3 (DOCX 28 KB) [file 787_2025_2648_MOESM3_ESM.docx]

**Mental Health Literacy Interventions for Female Adolescents: A Systematic Review and Meta-Analysis**

European Child & Adolescent Psychiatry

Emily R Arnold*^1^, Caitlin Liddelow^1^, Angie S X Lim^1^, Stewart A Vella^1^,

^1^ Global Alliance for Mental Health and Sport (GAMeS), School of Psychology, Faculty of the Arts, Social Sciences and Humanities, University of Wollongong, Northfields Avenue, Wollongong, NSW 2522

***Corresponding author:** Emily R Arnold - Global Alliance for Mental Health and Sport (GAMeS), School of Psychology, Faculty of the Arts, Social Sciences and Humanities, University of Wollongong, Northfields Avenue, Wollongong, NSW 2522. [era391@uowmail.edu.au](mailto:era391@uowmail.edu.au).

| **Category** | **Criteria** | **Study** | | | | | | | | | | | | |
| --- | --- | --- | --- | --- | --- | --- | --- | --- | --- | --- | --- | --- | --- | --- |
|  |  | Bella-Awusah et al.  2014 | Hassen et al. 2022 | Morgado et al.  2022 | Naylor et al. 2009 | Panza et al.  2022 | Patafio et al.  2021 | Perry et al.  2014 | Pinto-Foltza et al.  2011 | Russell et al.  2023 | Wei et al.  2022 | Wei et al.  2023 | Zare et al.  2021 | Total |
| **Reach** | Methods to identify target population | x | x | x | x | x | x | x | x | x | x | x | x | 12 |
|  | Exclusion Criteria (% excluded or characteristics) |  | x |  |  |  |  | x | x |  |  |  | x | 4 |
|  | Percentage of individuals who participate, based on valid denominator (not of volunteers who indicate interest) |  | *N/R females only | x | *N/R females only | *N/R females only | *N/R females only |  | x | *N/R females only |  | *N/R females only |  | 2 |
|  | Characteristics of participants compared to non-participants or target population |  |  |  |  |  |  |  |  |  |  |  |  | 0 |
|  | Use of qualitative methods to understand reach and/or recruitment |  |  |  |  |  |  |  |  |  |  |  |  | 0 |
| **Effectiveness** | Measure of primary outcome with or | x | x | x | x | x | x | x | x | x | x | x | x | 12 |
|  | w/o comparison to a public health goal |  |  |  |  |  |  |  |  |  |  |  |  |  |
|  | Measure of broader outcomes (e.g., measure of QoL or potential negative outcome) |  |  |  |  |  |  | x |  | x | x |  |  | 3 |
|  | Intention to treat analysis |  |  |  | x |  |  | x | x |  |  |  |  | 3 |
|  | Measure of Robustness across groups (e.g., moderation analysis, comparisons between different groups) |  | x |  |  |  | x |  |  |  |  |  |  | 2 |
|  | Measure of short-term attrition and short-term differential rates by patient characteristics or condition |  |  | N/A |  |  | x | x | x | *N/R females only |  | *N/R females only |  | 3 |
|  | Use of qualitative methods/data to understand outcomes |  |  |  |  | x |  |  | x |  |  |  |  | 2 |
| **Adoption - Setting** | Setting Exclusions (% or reasons) |  |  |  |  |  |  | x |  |  |  |  |  | 1 |
|  | Percent of settings approached that participate (valid denominator) |  |  |  |  | x |  | x |  | x | x | x |  | 5 |
|  | Characteristics of settings participating compared to either non-participants or some relevant resource data |  |  |  |  | x |  |  |  |  |  |  |  | 1 |
|  | Use of qualitative methods to understand adoption at setting level |  |  |  |  | x |  |  |  |  |  |  |  | 1 |
| **Adoption - Staff** | Exclusion of Staff (% or reasons) |  | N/A |  |  |  |  |  | x |  |  |  |  | 1 |
|  | Percent of staff invited that participated |  | N/A |  |  |  |  |  |  |  |  |  |  | 0 |
|  | Characteristics of staff participants vs. non-participating staff or typical staff |  | N/A |  |  |  |  |  |  |  |  |  |  | 0 |
|  | Use of qualitative methods to understand staff participation |  | N/A |  |  |  |  |  |  |  |  |  |  | 0 |
| **Implementation** | Adaptations made to intervention during study |  |  |  |  |  |  | x |  | x | x | x |  | 4 |
|  | Percent of perfect delivery or calls completed, etc. (e.g., adherence or consistency) |  |  |  |  |  |  |  | x |  |  |  |  | 1 |
|  | Cost Delivery (e.g., time & money) |  | x |  |  |  |  |  |  |  |  |  |  | 1 |
|  | Consistency of implementation |  |  |  |  |  |  |  |  |  |  |  |  | 0 |
|  | across staff, time, settings, subgroups |  |  |  |  |  |  |  |  |  |  |  |  |  |
|  | Theoretical Foundations |  | x | x |  | x |  |  | x |  |  |  |  | 4 |
|  | Qualitative methods to measure implementation |  |  | x |  |  |  |  |  |  |  |  |  | 1 |
| **Maintenance - Individual** | Measure of primary outcome (with or w/o comparison to a public health goal) at ≥ 6mo follow-up after final intervention | x |  |  |  |  |  | x |  |  |  | x |  | 3 |
|  | Measure of broader outcomes at follow-up (e.g., measure of |  |  |  |  |  |  | x |  |  |  |  |  | 1 |
|  | QoL or potential negative outcome) at follow-up |  |  |  |  |  |  |  |  |  |  |  |  |  |
|  | Robustness of long-term data |  |  |  |  |  |  |  |  |  |  |  |  | 0 |
|  | Measure of long-term attrition (%) and differential rates by patient characteristics or condition | x |  |  |  |  |  | x |  |  |  | x |  | 3 |
|  | Use of qualitative methods/data to understand long-term effects |  |  |  |  |  |  |  |  |  |  |  |  | 0 |
| **Maintenance - Setting** | If program is still ongoing at ≥ 6-month post study funding |  |  |  |  |  |  |  |  |  |  |  |  | 0 |
|  | If and how program was adapted long-term |  |  |  |  |  |  |  |  |  |  |  |  | 0 |
|  | Some measure/discussion of alignment to organization mission or |  |  |  |  |  |  |  |  |  |  |  |  | 0 |
|  | sustainability of business model |  |  |  |  |  |  |  |  |  |  |  |  |  |
|  | Use of qualitative methods data to understand setting level institutionalisation |  |  |  |  |  |  |  |  |  |  |  |  | 0 |

*Note:* Results are based on available female data.
